# Harnessing Machine Learning and Molecular Docking to Decode the Fatty Acid Dynamics in High-Altitude Yak Milk

**DOI:** 10.3390/ani16101477

**Published:** 2026-05-12

**Authors:** Chaoyun Yang, Yao Pan, Yi He, Ran Guan

**Affiliations:** Molecular Breeding Laboratory for Ruminants in Liangshan, Xichang University, Xichang 615000, China; xcc20220311@xcc.edu.cn (C.Y.); 15378490805@163.com (Y.P.); 15983842282@163.com (Y.H.)

**Keywords:** Muli yak milk, parity, functional fatty acids, machine learning, molecular docking

## Abstract

This study investigated the fatty acid profile of Muli yak (*Bos grunniens*) milk and its relationship with compositional parameters across different parities. Milk samples from second-, third-, and fourth-parity yaks were analysed for protein, fat, vitamins, minerals, and 37 fatty acids using gas chromatography. Statistical analyses included ANOVA, correlation analysis, principal component analysis (PCA), machine learning algorithms, and molecular docking. Parity significantly affected 15 components (*p* < 0.05), with third-parity milk showing the highest eicosapentaenoic acid (EPA, C20:5n3) and arachidonic acid (ARA, C20:4n6) concentrations. Among 134 significant correlations, calcium-ARA and ARA-EPA exhibited strong positive associations (|*r*| > 0.67). PCA explained 54.2% of the variance through three principal components, differentiating samples by parity. The optimal prediction models were ARA-XGBoost, EPA-Random Forest, ALA-GAM, and LA-SVM, with calcium and protein serving as key predictors. Molecular docking revealed that EPA-FABP2 had the lowest binding energy. These parity-related shifts in functional long-chain polyunsaturated fatty acids are meaningful for the nutritional value of yak milk (e.g., omega-3/omega-6 profile) and may also influence technological properties associated with milk fat composition (e.g., oxidative stability and processing behaviour), supporting parity-oriented quality evaluation and targeted utilisation of yak milk.

## Simple Summary

This study aimed to find a practical way to estimate these beneficial fatty acids using only the basic nutritional information that is already printed on everyday milk packaging, such as calcium, protein, fat, vitamin A, and sodium. We found that the number of times a yak has given birth influences the nutritional composition of its milk. Using this insight, we developed predictive models that can estimate health-relevant fatty acids without costly or complex laboratory testing. We also explored, at a basic molecular level, how these fatty acids may interact with proteins involved in fat processing in the body. Together, these findings offer a more accessible route for producers and consumers alike to better understand and make use of the nutritional value of yak milk.

## 1. Introduction

The *yak* (*Bos grunniens*), endemic to the Qinghai-Tibetan Plateau, is a highly prized livestock species with considerable pastoral significance. Muli yak milk, produced in the unique high-altitude ecological conditions of Sichuan Province at elevations exceeding 3000 m, exhibits distinctive nutritional attributes shaped by extreme environmental pressures. The hypoxic environment and specialised feed composition characteristic of alpine regions fundamentally alter metabolic pathways in mammary epithelial cells, resulting in milk fatty acid profiles that are distinctly divergent from conventional cattle breeds [1,2]. Such distinctive compositional patterns warrant specific scientific attention and demand tailored analytical approaches.

Research into the composition of milk is of critical importance for the development of the dairy industry, the promotion of consumer health, and the development of breeding strategies [3]. Conventional components (proteins, lipids, vitamins A and B, calcium, sodium) have long served as quality indicators in this field. However, it is now widely acknowledged that functional fatty acids have become a crucial evaluation parameter. As demonstrated in the relevant literature, polyunsaturated fatty acids (PUFAs) have been shown to have significant physiological functions, including anti-inflammatory effects, immune regulation, and cardiovascular protection [4,5]. Commercial dairy products generally disclose only conventional component content. This engenders information gaps, which in turn limit consumer understanding and fail to meet targeted fatty acid intake requirements.

Recent research findings have confirmed significant correlations between the composition of milk fatty acids and conventional components. Calcium concentration has been identified as a potentially influential factor in the process of fatty acid desaturation through the regulation of mammary epithelial cell enzymes [6]. Current prediction methodologies employ a range of techniques, including mid-infrared spectroscopy [7], dietary fatty acid intake modelling [8], and feed composition-based approaches [9]. However, these methods are predicated on conventional breeds such as Holstein, thus overlooking the metabolic specificities of high-altitude yaks and requiring sophisticated equipment. Machine learning algorithms, including XGBoost and Random Forest, represent a paradigm shift in predictive modelling by capturing nonlinear relationships and interactive effects among multiple variables without requiring specialised instrumentation, thereby enabling rapid prediction of functional fatty acids from readily accessible conventional milk parameters [10,11,12]. Such advantages render machine learning particularly suitable for resource-constrained settings and breed-specific applications.

The fatty acid composition of milk is influenced by several factors, including breed, feeding regimen, parity, and lactation stage [13]. Parity significantly influences metabolic maturity and mammary gland development; primiparous animals exhibit incomplete mammary epithelial differentiation and physiologically immature enzyme systems, thereby producing milk with substantially different fatty acid profiles compared to multiparous animals [14]. The second, third, and fourth parities represent the optimal metabolic window for studying compositional stability and functionality in yak milk, as these animals have attained full mammary gland maturation while remaining within peak lactational efficiency [15,16,17].

The present study systematically investigated Muli yak milk to: (1) identify compositional variations across parities; (2) examine component relationships through correlation analysis and PCA; (3) develop machine learning-based predictive models for functional fatty acids from conventional indicators; and (4) investigate fatty acid–protein interactions using molecular docking techniques to elucidate mechanistic pathways underlying compositional variation.

## 2. Materials and Methods

### 2.1. Milk Sample Collection

Milk samples were collected from Muli yaks using modified manual milking techniques by dairy hygiene standards (parities 2 [n = 11], 3 [n = 11] and 4 [n = 8]). Personnel were subjected to standard hygiene protocols, which included the disinfection of their hands with 0.5% chlorhexidine gluconate and the donning of disposable coveralls, nitrile gloves, and face masks. The two-stage pre-milking stimulation method involved the following steps: firstly, udder cleansing with warm water (40–42 °C) containing 0.1% sodium dodecyl sulfate, and secondly, teat disinfection using cotton pads soaked in 70% isopropyl alcohol. Milk was collected into pre-sterilised steel containers, filtered through 0.45 μm sterile membranes, and cooled to 4 ± 0.5 °C within 10 min. Subsequently, 50 mL samples were collected into sterile, enzyme-free tubes, transported, and stored at 4 °C pending analysis.

### 2.2. Quantification of Milk Components

Systematic quantitative analyses were performed for protein, multi-class veterinary drug residues, sodium (Na), calcium (Ca), vitamin A (VA), vitamin D (VD), and fatty acid composition. The protein content was determined using the Kjeldahl method. The analysis of multi-class veterinary drug residues was conducted by the provisions outlined in CFI/QT-B0005-2022. Extraction and concentration utilised an automated nitrogen evaporator (N1-50, Shanghai Yiyao Instrument Technology Co., Shanghai, China), with analysis by gas chromatography–mass spectrometry (GC-MS) or liquid chromatography–mass spectrometry (LC-MS) [18]. The determination of the Na and Ca concentrations was accomplished through the utilisation of inductively coupled plasma optical emission spectrometry (ICP-OES; Optima 8300, PerkinElmer, Hong Kong, China), a method that involved the digestion of samples via microwave heating. The fatty acid composition was established using gas chromatography after methylation. The quantification of retinol was conducted using high-performance liquid chromatography (HPLC; 1260, Agilent Technologies, Santa Clara, CA, USA) with UV detection. The analysis was performed using a high-performance liquid chromatography (HPLC) method. It is essential to note that all studies were conducted using pre-calibrated instruments, thereby ensuring the accuracy and reproducibility of the results obtained. The individual fatty acids were labelled FA1-FA37 (see Table S1), and 156 screened veterinary drugs were listed in Table S2.

### 2.3. Data Analysis

#### 2.3.1. Analysis of Variance

One-way analysis of variance was performed using the R statistical computing environment (v4.3.1) with various statistical packages. Raw data were extracted and standardised using the “openxlsx” package (v4.2.5). Statistical assumption validation involved Shapiro–Wilk normality testing and Levene’s homogeneity of variances test, using the “rstatix” package (v0.7.2) and the “car” package (v3.1-2). Quantile–quantile plots were generated with the “ggplot2” package (v3.4.3) for visual data distribution evaluation. ANOVA modelling employed a linear model (Formula: y~Parity), calculating F-statistics and *p*-values via Type III sums of squares. When significant between-group differences were detected (α = 0.05), Tukey’s Honestly Significant Difference post hoc testing was conducted. Visualisation was performed using the “ggpubr” package (v0.5.0). Analyses included data from parities 2, 3, and 4, with the following significance levels: * *p* < 0.05; ** *p* < 0.01; and *** *p* < 0.001.

#### 2.3.2. Correlation Analysis

Pearson’s product-moment correlation coefficients were calculated using the “Hmisc” package (v4.8-5), which generated correlation matrices and significance *p*-values from Bonferroni-corrected two-sided tests. Independent correlation analyses were conducted for each parity group using parallel processing to identify subgroup-specific patterns. Visualisation employed a hybrid graphical syntax with base correlation matrix heatmaps constructed using the “ggcor” package (v1.1.1), which incorporates built-in clustering algorithms for variable ordering optimisation. The graphical rendering was achieved by employing the ggcorrplot package (v0.1.5), which utilised gradient colour scales to map correlation coefficient magnitudes. Asterisks were used to indicate statistical significance (* *p* < 0.05; ** *p* < 0.01; *** *p* < 0.001).

#### 2.3.3. Principal Component Analysis (PCA)

The present study established a standardised PCA workflow using the R (v4.3.1) statistical computing environment. Standardised dimensionality reduction was performed with scale normalisation, thereby mitigating variable scale influences. The variance explained by each principal component was calculated using the eigen decomposition method. The optimal principal components were determined using a cumulative variance threshold of 80% or greater. The analysis employed the “ggplot2” (v3.4.3), “dplyr” (v1.1.2), “tidyr” (v1.3.0), and “factoextra” (v1.0.7) packages.

### 2.4. Model Construction and Optimisation

An efficient multi-model comparative analysis system was constructed using the R platform (v4.3.1), integrating 15 specialised packages for comprehensive data analysis and modelling. The data management process utilised the “openxlsx” (v2.4.0) and “caret” (v6.0-92) packages for training and evaluating the model, respectively. The machine learning algorithms included “e1071” (v1.7-9) for SVM [19], “glmnet” (v4.1-4) for regularised regression, “randomForest” (v4.7-1.1) [20], “mgcv” (v1.8-40) for GAMs [21], “xgboost” (v1.6.0.1), “nnet” (v7.3-17) for neural networks, and “pls” (v2.8-0) for PLS regression. The visualisation employed the “ggplot2” (v3.3.6), “gridExtra” (v2.3), “Metrics” (v0.1.4), “reshape2” (v1.4.4), and “tidyverse” (v1.3.2) suite for data manipulation and workflow management.

#### 2.4.1. Linear and Polynomial Regression Models

The linear regression model utilised in this study employed Ordinary Least Squares (OLS) for parameter estimation. The model is formulated as follows: *Y* = *β*_0_ + *β*_1_*Ca* + *β*_2_*Pro* + *β*_3_*VA* + *β*_4_*FAT* + *β*_5_*Na* + *ε*, where *Y* is the response variable (including specific fatty acids LA, ALA, ARA, and EPA); *β*_0_ is the intercept. The regression coefficients, denoted by *β*_1_ to *β*_5_, correspond to the variables Ca, Pro, VA, FAT, and Na, respectively. The random error term, designated as ε, is assumed to follow a normal distribution with a mean of zero and a variance of *σ^2^*. A quadratic polynomial regression model was constructed to address potential multicollinearity by introducing quadratic terms (*X_i_*^2^) for each predictor variable. The model is expressed as follows: *Y* = *β*_0_ + *Σβᵢ*_1_*Xᵢ* + *Σβᵢ*_2_*Xᵢ*^2^ + *ε*, where *X_i_* represents the standardised predictor variables. Additionally, a polynomial regression model incorporating first-order interaction terms was used to capture synergistic effects between variables. This model was formulated as follows: *Y* = *β*_0_ + *Σβᵢ*_1_*Xᵢ* + *ΣΣβᵢⱼXᵢXⱼ* + *ε* (where *i* ≠ *j*). Incorporating interaction terms has significantly enhanced the model’s capacity to accommodate intricate non-linear relationships. During the training phase of the model, the variance inflation factor was utilised to identify multicollinearity. Variables with a variance inflation factor more significant than 10 were excluded to improve the model’s structural integrity.

#### 2.4.2. Non-Linear Regression Models

Support vector regression (SVR) [19] utilised a radial basis function (RBF) kernel for non-linear relationships, with optimised hyperparameters (γ = 0.215, C = 4.64, and ε = 0.1) determined through a grid search. The enhanced prediction stability of random forest (RF) [20] is attributable to the multiple decision trees with optimised parameters, specifically setting ntree = 500, mtry = 2, and nodesize = 5. Generalised additive models (GAMs) [21] are a class of statistical models that utilise cubic regression splines to capture non-linear effects. The smoothing parameters of these models are estimated using Maximum Likelihood Estimation (MLE). This method automatically selects parameters to balance the model’s goodness of fit and the risk of overfitting.

#### 2.4.3. Ensemble and Deep Learning Models

Gradient boosting (XGBoost, R package v1.6.0.1) is a machine learning algorithm that employs an efficient decision tree framework with optimised parameters. These include the learning rate, maximum tree depth, and subsampling ratios. The implementation of early stopping prevents overfitting. These include the learning rate, maximum tree depth, and subsampling ratios. The implementation of early stopping prevents overfitting. The neural network (NNET) employed a feed-forward architecture that was optimised through 10-fold cross-validation, resulting in 5 input nodes, 10 hidden layer nodes, and one output node. Multicollinearity was addressed using partial least squares (PLS) regression, which employed dimensionality reduction. The optimal number of latent variables was determined through 10-fold cross-validation, selecting ncomp = 3. The construction process evaluated the applicability and predictive performance of each model, thereby establishing robust foundations for subsequent evaluation and analysis.

#### 2.4.4. Model Evaluation Criteria

The performance of the models was evaluated using four standardised metrics to facilitate a comparison across multiple models. Root mean square error (RMSE) was utilised as the primary evaluation metric, with lower values denoting higher prediction accuracy. The mean absolute error (MAE) is a metric of model robustness to outliers, with smaller values indicating better stability. The coefficient of determination (R^2^) was utilised to quantify the proportion of explained variance, with values approaching 1 indicating enhanced explanatory power. The mean absolute percentage error (MAPE) was used as a metric to assess the relative prediction accuracy, with lower values indicating superior performance. In the context of a systematic evaluation, a comprehensive ranking of all metrics was conducted for each model, with primary emphasis placed on RMSE, complemented by R^2^ assessment outcomes.

### 2.5. Molecular Docking and Molecular Dynamics Simulation

The present study employed molecular docking techniques using AutoDock Vina (version 1.2.2) [22] to evaluate the interactions between functional fatty acids and target proteins. Four functional fatty acids were obtained from the PubChem database. ALA (CID: 5280934), LA (CID: 5280450), EPA (CID: 446284), and ARA (CID: 444899) are the relevant cases in this instance. The target proteins selected for investigation included apolipoprotein A1 (APOA1, PDB: 2A01), apolipoprotein A4 (APOA4, PDB: 3S84), CD36 (PDB: 5LGD), and fatty acid binding protein 2 (FABP2, PDB: 1KZW), obtained from the RCSB Protein Data Bank and AlphaFold Database. The pre-processing stage involved converting the PDBQT structure format using an MMFF94 force field [23], removing crystal water molecules, and adding polar hydrogen atoms. The docking process was conducted within a 30 Å cubic search space, with a grid spacing of 0.05 nm. The results were evaluated using energy scores and subsequently visualised using PyMOL (v3.1.4.1).

## 3. Results

### 3.1. Differential Analysis Identifies Key Milk Components Across Lactation Parities

**Figure 1:**
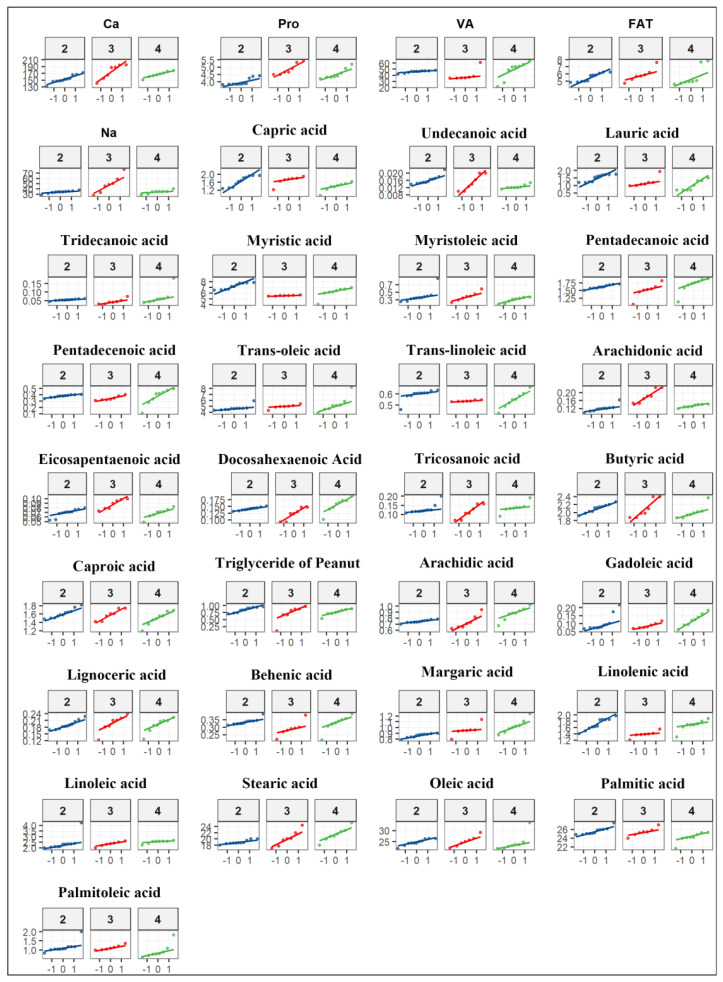
QQ plots for normality tests of different detection indicators. Three colours represent different parities: the blue curve denotes the second parity, the red curve denotes the third parity, and the green curve denotes the fourth parity.

In the present study, rigorous screening confirmed the absence of veterinary drug residues in all collected milk samples. To validate the assumptions for parametric statistical analysis, preprocessing included the Shapiro–Wilk test for normality and Levene’s test for homogeneity of variances. The results indicated that the acquired data met the fundamental assumptions required for analysis of variance (Figure 1).

The F-test results (Table 1 and Table S3) revealed statistically significant differences across lactation parities for 15 key components, including Ca, Na, and Pro. Specifically, substantial disparities were identified for the following fatty acids: decanoic acid (C10:0), ARA (C20:4n6), EPA (C20:5n3), and heneicosanoic acid (C21). The following acids were identified: undecanoic acid (C11:0), arachidic acid (C20:0), lauric acid (C12:0), heptadecanoic acid (C17:0), ALA (C18:3), stearic acid (C18:0), palmitic acid (C16:0), and myristic acid (C14:0). These components exhibited substantial disparities (*p* < 0.05) among a minimum of two parity groups, thus necessitating post hoc multiple comparisons.

**Figure 2:**
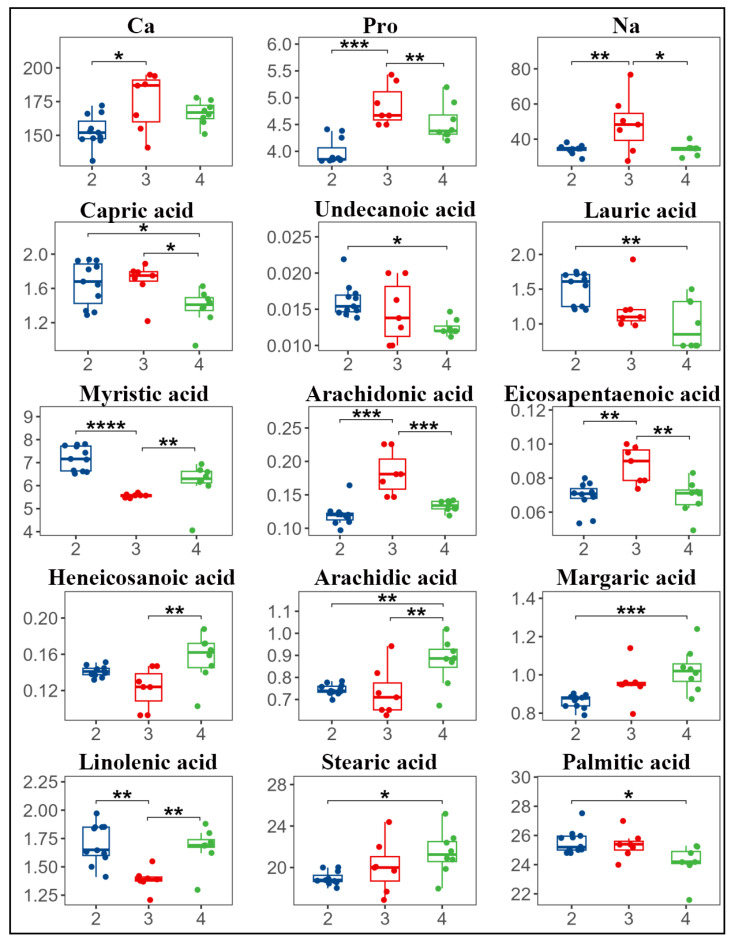
Box plots of multiple tests for each indicator (* *p* < 0.05,** *p* < 0.01,*** *p* < 0.001, **** *p* < 0.0001). Three colours represent different parities: the blue curve denotes the second parity, the red curve denotes the third parity, and the green curve denotes the fourth parity.

A subsequent multiple comparison analysis revealed significant differences in amino acid components between different parities (Figure 2 and Table S3). Specifically, a comparison was made between Parity 2 and Parity 3, with the results indicating a statistically significant difference in calcium content, with Parity 2 demonstrating lower levels of Ca (*p* < 0.05). The concentrations of protein, Na, ARA, and EPA were found to be significantly lower in Parity 2 (*p* < 0.01). In contrast, the levels of myristic acid (C14:0) and ALA (C18:3) were found to be significantly higher in parity 2 compared to Parity 3 (*p* < 0.01).

A comparison was made between Parity 2 (n = 11) and Parity 4 (n = 8), with a focus on the levels of decanoic acid (C10:0), undecanoic acid (C11:0), and palmitic acid (C16:0). The results indicated that Parity 2 exhibited significantly higher levels of these acids (*p* < 0.05). Additionally, lauric acid (C12:0) and myristic acid (C14:0) demonstrated highly significant increases (*p* < 0.01). In contrast, stearic acid (C18:0) exhibited a significant decrease (*p* < 0.05), and arachidic acid (C20:0), protein, and heptadecanoic acid (C17:0) demonstrated a highly significant reduction in Parity 2 (*p* < 0.01).

A comparison was made between Parity 3 (n = 7) and Parity 4. Specifically, the study focused on the analysis of decanoic acid (C10:0) levels, with the results indicating a notable increase in its presence in Parity 4. Na, EPA, and ARA exhibited highly significant increases in Parity 4 (*p* < 0.01). In contrast, arachidic acid (C20:0) showed a significant decrease (*p* < 0.05), and heneicosanoic acid (C21:0) and ALA (C18:3) demonstrated highly substantial reductions in Parity 4 compared to Parity 3 (*p* < 0.01).

### 3.2. Correlation Analysis Identifies Key Milk Components

**Figure 3:**
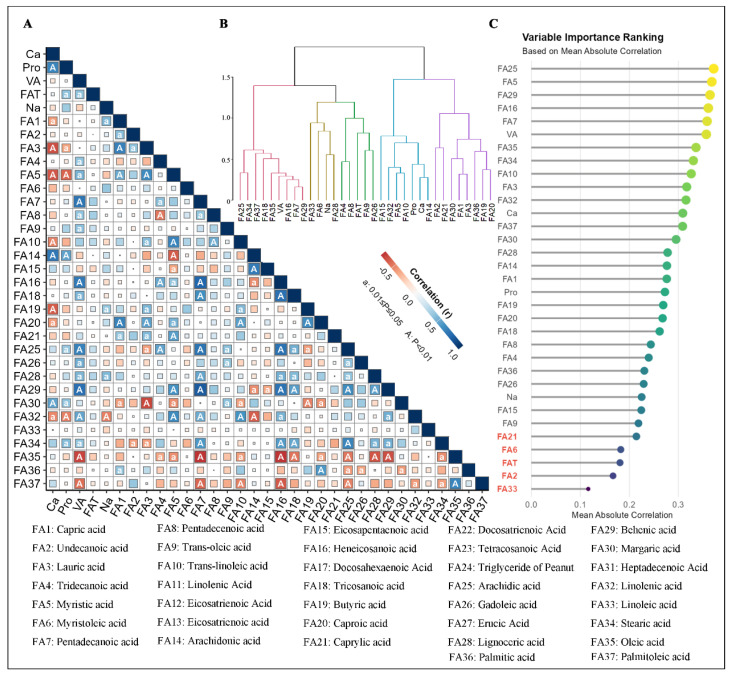
Correlation analysis, cluster analysis, and variable importance ranking of multiple indicators. Panel (**A**) displays a Pearson correlation coefficient matrix, presenting the inter-indicator relationships. Therein, deep blue indicates a strong positive correlation, deep red indicates a strong negative correlation, and white indicates a weak correlation. The size of the rectangular blocks is positively correlated with the absolute value of the correlation coefficient; larger blocks represent larger absolute values of the correlation coefficients. Panel (**B**) is a hierarchical clustering dendrogram based on correlation coefficients, which systematically reveals the clustering relationships among indicators. Panel (**C**) is a variable importance assessment diagram based on mean absolute correlation coefficients, intuitively reflecting the contribution of each indicator to the overall sample variation.

The present study identified a total of 134 significant correlations between variables (Figure 3A, marked as “A” or “a”). At a significance level of α = 0.01, the top 20 absolute correlation coefficients (|cor| > 0.67) are enumerated in Table 2. The relationships in question exhibit a high degree of significance and strong correlation. Specifically, heneicosanoic acid (C21:0) showed highly significant positive correlations (cor > 0.67, *p* < 0.01) with pentadecanoic acid (C15:0), tricosanoic acid (C23:0), arachidic acid (C20:0), behenic acid (C22:0), and Vitamin A. Concurrently, heneicosanoic acid (C21:0) exhibited a highly significant negative correlation (cor = −0.67, *p* < 0.01) with oleic acid (C18:1). In addition, arachidic acid (C20:0) exhibited highly significant positive correlations (cor > 0.67, *p* < 0.01) with pentadecanoic acid (C15:0), vitamin A, and stearic acid (C18:0). Similarly, pentadecanoic acid (C15:0) demonstrated highly significant positive correlations (cor > 0.67, *p* < 0.01). Behenic acid (C22:0) and vitamin A demonstrated a highly significant positive correlation (cor > 0.67, *p* < 0.01). Similarly, behenic acid (C22:0) exhibited a highly significant positive correlation (r > 0.67, *p* < 0.01) with vitamin A. Conversely, oleic acid (C18:1) demonstrated a highly significant negative correlation (r = −0.67, *p* < 0.01) with vitamin A and pentadecanoic acid (C15:0).

In addition to the observed correlations, a series of highly significant positive relationships (cor > 0.67, *p* < 0.01) were identified. These relationships were observed between Na and ARA, between ARA and EPA (C20:5n3), between capric acid (C10:0) and lauric acid (C12:0), and within caproic acid (C6:0). Finally, a highly significant negative correlation was demonstrated by lauric acid (C12:0) and margaric acid (C17:0) (cor = −0.67, *p* < 0.01). In summary, these results reveal strong correlations among multiple variables, providing a crucial basis for subsequent research into the functions of these compounds in biological processes. A more profound comprehension of these substantial correlations may present novel perspectives and directions for scientific research and its applications.

The present study employed cluster analysis to explore the component interrelationships, a process that revealed four main categories (Figure 3B). The following short- and medium-chain fatty acids were included: caproic acid (C6:0), butyric acid (C4:0), lauric acid (C12:0), capric acid (C10:0), palmitic acid (C16:0), margaric acid (C17:0), caprylic acid (C8:0), and undecanoic acid (C11:0). These acids possess high biological activity and participate in cellular energy metabolism and membrane function. Long-chain polyunsaturated fatty acids comprised ARA (C20:4n6), calcium, protein, trans-LA (C18:2n6t), myristic acid (C14:0), ALA (C18:3), and EPA (C20:5n3). These components are of pivotal significance in the realm of cell signalling, inflammatory responses, and the regulation of cellular function. As indicated by the current literature, the unsaturated long-chain fatty acids that have been identified include gadoleic acid (C20:1), trans-oleic acid (C18:1n9t), fat, pentadecenoic acid (C15:1), tridecanoic acid (C13:0), lignoceric acid (C24:0), sodium, myristoleic acid (C14:1), and LA (C18:2). The evidence suggests that these are linked to lipid metabolism and cardiovascular health. Long-chain saturated fatty acids and vitamins contained oleic acid (C18:1), stearic acid (C18:0), behenic acid (C22:0), pentadecanoic acid (C15:0), heneicosanoic acid (C21:0), vitamin A, tricosanoic acid (C23:0), palmitoleic acid (C16:1), and arachidic acid (C20:0), affecting lipid storage, antioxidant activity, and growth regulation. The variable importance results from the mean absolute correlation analysis were consistent with the clustering results (Figure 3C), systematically revealing the fatty acid composition and providing a foundation for subsequent research.

### 3.3. Identification of Key Milk Components via Principal Component Analysis

**Figure 4:**
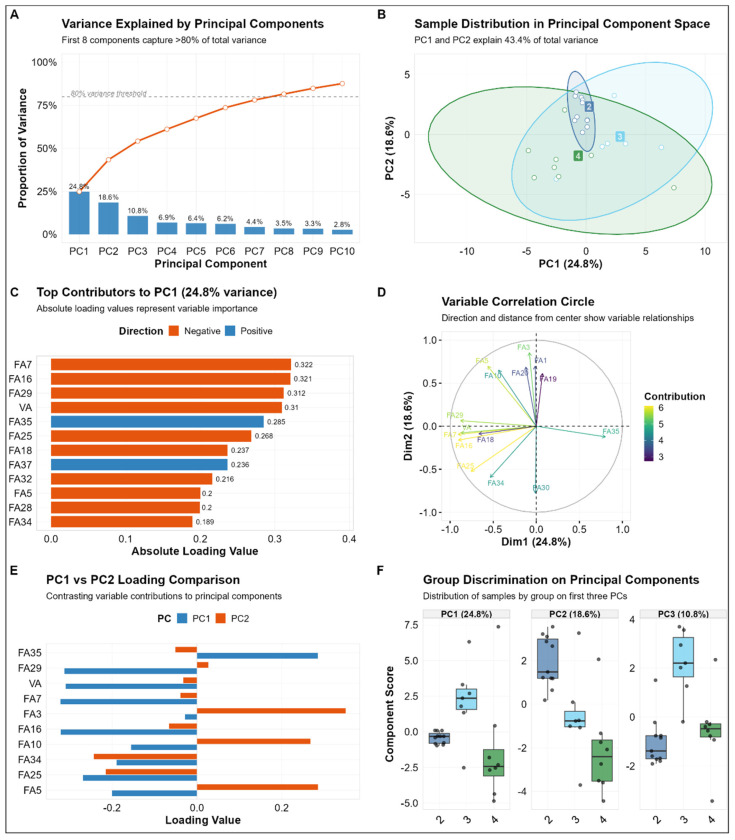
Principal component analysis. Panel (**A**) shows a bar chart of variance contribution rates for principal components, demonstrating that the cumulative variance contribution rate of the first 8 principal components exceeds 80%. Panel (**B**) displays the distribution of samples in the principal component space constituted by PC1 and PC2, where different colours represent different parities, illustrating the clustering characteristics of samples in the principal component space. Panel (**C**) is a variable contribution ranking plot for PC1, where orange bars indicate negative contributions and blue bars indicate positive contributions; the values represent the magnitude of the absolute loading values. Panel (**D**) is a variable correlation circle plot, showing the correlative relationships between each variable and PC1 and PC2, as well as their contribution levels; variables that are further from the centre indicate greater contributions to the principal components. Panel (**E**) is a comparative plot of loading values for PC1 and PC2, where blue bars represent PC1 loading values and orange bars represent PC2 loading values, elucidating the relative contribution differences in different variables to the two principal components. Panel (**F**) is a box plot showing the distribution of component scores for samples from different parities on the first three principal components, used to assess the extent of the influence of parity on milk composition variation.

The present study’s findings indicate that eight principal components (PC1–PC8) were retained, as their cumulative contribution rate exceeded 80%. The individual variance contributions for the first ten calculated principal components were 24.8%, 18.6%, 10.8%, 6.9%, 6.4%, 6.2%, 4.4%, 3.5%, 3.3%, and 2.8%, respectively (Figure 4A). Among these, PC1 and PC2 exhibited the highest contribution rates, at 24.8% and 18.6%, respectively, suggesting that these two principal components play a dominant role in explaining the data variance. Furthermore, the distribution of samples from different parities across PC1 and PC2 exhibited clear clustering patterns (Figure 4B), indicating that parity significantly influences the variation in milk composition.

To further elucidate the contribution of each variable to the principal components, we examined the factor loadings (Figure 4C). The study’s findings indicated that unsaturated fatty acids (e.g., oleic acid, palmitoleic acid, and ARA) exhibited negative loadings on the principal components. In contrast, saturated fatty acids (e.g., pentadecanoic acid, heneicosanoic acid, and behenic acid) demonstrated positive loadings. The correlation circle plots (Figure 4D,E) further elucidate the contribution patterns of variables to Principal Components 1 (PC1) and 2 (PC2). PC1 exhibited a robust correlation with the loadings of unsaturated fatty acids (oleic acid, palmitoleic acid, and ARA). In contrast, PC2 demonstrated a predominant influence from saturated fatty acids, myristic acid, and trans-LA. This finding suggests a close relationship between the variation in milk composition and the degree of fatty acid saturation.

A subsequent analysis of the scores of different parity groups on PC1, PC2, and PC3 revealed distinct differences between parities. Specifically, based on the average scores, the ranking of parities along PC1 was Parity 3 > Parity 2 > Parity 4; along PC2, it was Parity 2 > Parity 3 > Parity 4; and along PC3, it was Parity 3 > Parity 4 > Parity 2 (Figure 4F). These results further corroborate the notion that parity is a substantial factor influencing variations in milk composition. Furthermore, the distinct scoring patterns exhibited by different parity groups along the principal components indicate the dynamic nature of compositional changes across parities.

### 3.4. Machine Learning Algorithms: Construct Predictive Models for Key Fatty Acid Indicators

Integrating results from differential analysis, correlation analysis, and PCA, we precisely identified and selected four key functional fatty acids from the complex dataset: ARA (C20:4n6, designated FA14), EPA (C20:5n3, designated FA15), ALA (C18:3, designated FA32), and LA (C18:2, designated FA33). These fatty acids were selected as core indicators for machine learning modelling. This selection was based on two factors: their statistical significance and their complementary biological functions and representative characteristics.

**Figure 5:**
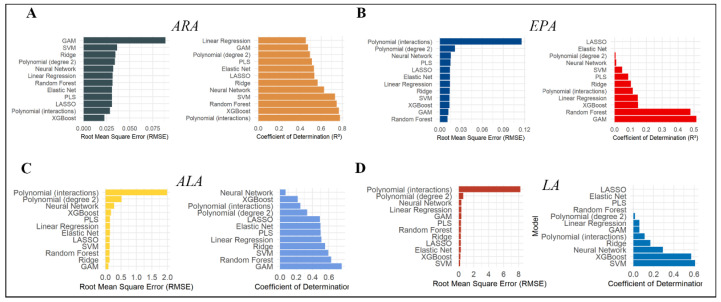
Optimal model screening scores for four variables. Panels (**A**–**D**) display the prediction model evaluation for four fatty acid components: ARA (arachidonic acid), EPA (eicosapentaenoic acid), ALA (alpha-linolenic acid), and LA (linoleic acid), respectively. The left portion of each panel presents a ranking of different machine learning models based on Root Mean Square Error (RMSE), including polynomial interactions, stepwise regression, neural network, linear regression, random forest, and support vector machine; the right portion displays the corresponding coefficient of determination (R^2^) values. Lower RMSE and higher R^2^ values indicate superior model predictive performance.

A thorough evaluation of 12 machine learning algorithms was conducted, leading to the identification of the optimal predictive models for ARA, EPA, ALA, and LA. The identified models include XGBoost, Random Forest (RF), Generalised Additive Model (GAM), and Support Vector Machine (SVM). The performance metrics for these models were as follows: The root mean square error (RMSE) values were 0.022, 0.011, 0.097, and 0.185, and the coefficients of determination (R^2^) were 0.769, 0.478, 0.760, and 0.609, respectively (Table 3 and Figure 5). These results provide substantial validation of the reliability and accuracy of the selected models in predicting the dynamic changes in these key fatty acids.

For the optimal XGBoost model predicting ARA, the model equation is represented as $\overset{\wedge }{y}=\sum_{i=1}^{88}\alpha_{i}h_{i}(X)$. The loss function is defined as $L(y,\overset{\wedge }{y})=\frac{1}{88}\sum_{j=1}^{88}{\left(y_{j}-\overset{\wedge }{y_{j}}\right)}^{2},$ where $\overset{\wedge }{y}$ represents the predicted value, $\alpha_{i}$ is the weight assigned to each base learner, and $h_{i}(X)$ denotes the k-th base learner. The loss function aims to minimise the difference between the actual values and the predicted values ($\overset{\wedge }{y}$). Parameters including max_depth = 5, learning rate (eta) = 0.1, subsample = 1, and colsample_bytree = 1 controlled the complexity of each base learner. According to this model (Table 4), the top two most essential input variables were Ca (importance score: 0.44) and Pro (importance score: 0.273).

The key hyperparameters for the Random Forest model predicting EPA were set as follows: ntree = 300, mtry = 1, and nodesize = 5. The optimal model formula is represented as $\overset{\wedge }{y}=\frac{1}{300}\sum_{k=1}^{300}T_{300}(X)$. According to the variable importance analysis for this model (Table 4), the two most important predictors were Ca (Ca, importance score: 0.45) and Pro (Pro, importance score: 0.27).

For the GAM predicting ALA, the optimal model equation was determined as y~s(Ca, k = 5) + s(Pro, k = 5) + s(VA, k = 5) + s(FAT, k = 5) + s(Na, k = 5). In this formulation, y represents the response variable (ALA), and s (variable, k = 5) denotes a smooth function of the respective predictor variable (Ca, Pro, VA, Fat, Na), estimated using a basis dimension (k) of 5. Based on the variable importance analysis for this model (Table 4), the top two most influential predictors were FAT and Pro.

For the SVM model predicting LA, a linear kernel function was utilised. The cost parameter was set to 0.1, and epsilon was set to 0.01. Cross-validation resulted in an RMSE of 0.37, with a standard deviation for the cross-validated RMSE of 0.41. Variable importance analysis (Table 4) identified the two most significant predictors as Na (sodium, importance: 2.35) and VA (vitamin A, importance: 2.35). The optimal model equation is represented as $f(x)=\sum_{i=1}^{20}(y_{i}\cdot \alpha_{i}\cdot K(x_{i},x))+b$. In this formulation, $K(x_{i},x)$ was the polynomial kernel function, α_i_ represents the Lagrange multipliers, y_i_ corresponds to the label of the respective sample, and b is the bias term.

### 3.5. Molecular Docking

**Figure 6:**
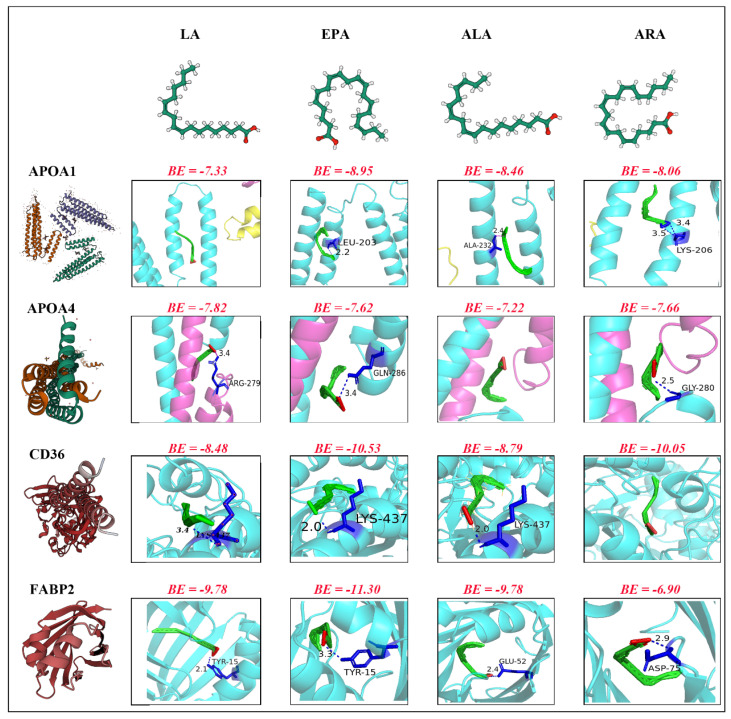
Molecular docking results. The figure displays the molecular docking results between four fatty acid components (LA, EPA, ALA, and ARA) and four proteins (APOA1, APOA4, CD36, and FABP2). The left panel shows the three-dimensional structures of the four proteins, while the top panel presents the molecular structures of the four fatty acids. The binding energy (BE) value (kcal/mol) is indicated above each docking model. Protein structures are illustrated in cyan, magenta, and other colours representing different structural domains, with fatty acid molecules depicted as green ball-and-stick models and relevant residues highlighted in indigo, green, and yellow. Lower binding energy values indicate stronger binding affinity between fatty acids and proteins.

Molecular docking analysis evaluated the binding affinity of four functional fatty acids (LA, EPA, ALA, ARA) towards four candidate protein targets (APOA1, APOA4, CD36, FABP2). The binding modes and interaction energies simulated using Autodock Vina v.1.2.2 indicated that all tested fatty acids successfully occupied the hydrophobic binding pockets of their respective target proteins (Figure 6). The primary binding driving force was identified as strong electrostatic interactions supplemented by hydrogen bond formation, a pattern observed in most ligand–protein complexes.

For the APOA1 protein (Figure 6), apart from LA (Binding Energy, BE = −7.33 kcal/mol), which bound primarily via electrostatic interactions, EPA (BE = −8.95 kcal/mol), ALA (BE = −8.46 kcal/mol), and ARA (BE = −8.06 kcal/mol) all additionally formed hydrogen bonds with residues LEU-203 (2.2 Å), ALA-232 (2.4 Å), and LYS-206 (3.4 Å), respectively. Regarding the APOA4 protein, the pattern differed: ALA (BE = −7.22 kcal/mol) primarily relied on electrostatic binding, while LA (BE = −7.82 kcal/mol), EPA (BE = −7.62 kcal/mol), and ARA (BE = −7.66 kcal/mol) all formed hydrogen bonds with residues ARG-279 (3.4 Å), GLN-286 (3.4 Å), and GLY-280 (2.5 Å), respectively.

In interactions with the CD36 protein (Figure 6), ARA (BE = −10.05 kcal/mol) binding was mainly mediated by strong electrostatic forces. In contrast, LA (BE = −8.48 kcal/mol), EPA (BE = −10.53 kcal/mol), and ALA (BE = −8.79 kcal/mol), besides their electrostatic interactions, all formed hydrogen bonds with the LYS-437 residue (bond lengths: 3.4 Å, 3.4 Å, and 2.0 Å, respectively). For the FABP2 protein, all four fatty acids employed both electrostatic attraction and hydrogen bonding forces for binding. Among these, EPA demonstrated the most favourable binding energy (BE = −11.30 kcal/mol, H-bond with TYR-15 at 3.3 Å), followed by LA (BE = −9.78 kcal/mol, H-bond with TYR-15 at 2.1 Å). ALA had a comparatively weaker binding energy (BE = −6.90 kcal/mol) but formed hydrogen bonds with GLU-52 (3.4 Å) and ASP-75 (2.9 Å). The interaction pattern for ARA with FABP2 was analogous, also involving hydrogen bonding.

## 4. Discussion

### 4.1. Effect of Parity on Milk Composition of Muli Yaks

The present study demonstrates that parity exerts a substantial influence on the composition of Muli *yak* milk, affecting the levels of various fatty acids, including ALA, EPA, LA, ARA, and calcium. Mid-parity yaks, particularly third parity, exhibited peak polyunsaturated fatty acids (PUFAs), such as eicosapentaenoic acid (EPA) and arachidonic acid (ARA), alongside increased C21:0 and C20:0. The preliminary findings have indicated that early parity milk samples have shown elevated levels of medium-chain saturated fatty acids (MCFAs), including C12:0 and C14:0. These are recognised as indicators of dairy quality and nutritional value. These patterns are indicative of metabolic adaptations that occur across lactation cycles, driven by the maturation of the mammary gland and changes in hormonal regulation. A substantial body of research has demonstrated that the lactation stage and parity have a significant impact on lipid biosynthesis in various species [17,24]. This phenomenon can be attributed to the altered expression of key enzymes, such as fatty acid desaturase 1 (FADS1) and acetyl-CoA carboxylase (ACC), which regulate desaturation pathways [25]. Furthermore, calcium levels exhibit variation according to parity, which is consistent with altered mineral transport systems involving PTHrP or CaSR [26].

It has been demonstrated that lactation progression is capable of inducing compositional changes, which have been shown to affect nutritional value and health benefits. As the onset of lactation is characterised by elevated levels of polyunsaturated fatty acids (PUFAs), the ratio of monounsaturated to saturated fatty acids undergoes a corresponding increase as lactation progresses [27]. Colostrum has been found to contain high-molecular-weight TAGs, while mature milk has been found to contain more medium-chain fatty acids [28,29]. As demonstrated in the relevant human studies [30,31], analogous patterns have been observed in premature infant milk. It is evident that maternal dietary intake, particularly the consumption of PUFAs during pregnancy and the postpartum period, exerts a substantial influence on the composition of milk [32].

These parity-driven compositional dynamics carry direct implications for the yak milk production sector. The identification of the third parity as the optimal window for PUFA enrichment provides an evidence-based criterion for herd management strategies: prioritising third-parity yaks in milking rotations could maximise the functional fatty acid yield of bulk milk, thereby enhancing the nutritional premium of yak dairy products destined for health-conscious consumer markets. Furthermore, the elevated MCFA levels observed in early-parity milk suggest that milk from second-parity yaks may be preferentially allocated to infant formula or specialised nutritional products, given the established role of MCFAs in rapid energy provision and antimicrobial activity [33]. From a breeding perspective, the significant inter-parity variation in EPA and ARA concentrations indicates that parity-associated metabolic maturity could serve as a phenotypic selection criterion in breeding programmes aimed at improving the functional lipid profile of yak milk. Incorporating parity-specific fatty acid data into genomic selection indices would enable breeders to identify sires whose daughters exhibit accelerated mammary metabolic maturation and sustained PUFA biosynthesis capacity across successive lactations [34].

In comparison with other dairy species and high-altitude breeds, the parity-dependent PUFA enrichment pattern observed in Muli yak milk exhibits both convergent and divergent features. Holstein and Jersey cattle display relatively modest parity effects on long-chain PUFA concentrations, which are primarily attributable to the homogenising influence of total mixed ration (TMR) feeding systems that buffer metabolic variability [35]. In contrast, extensively managed breeds such as the Laoshan goat [13] and Italian goat breeds [36] demonstrate more pronounced lactation-stage effects on fatty acid composition, although parity as an independent variable has received limited systematic investigation in these species. Notably, yak milk from the Qinghai-Tibetan Plateau has been reported to contain higher baseline levels of omega-3 PUFAs compared to lowland cattle breeds, a phenomenon attributed to the predominance of alpine Poaceae and Cyperaceae species that are rich in α-linolenic acid in natural grazing pastures [37]. The present findings extend this body of knowledge by demonstrating that the inter-parity amplitude of EPA and ARA variation in yak milk (approximately 1.5–2.0-fold between second and third parities) substantially exceeds that reported for Holstein cows under controlled feeding conditions (typically <1.2-fold) [38], suggesting that the metabolic plasticity of high-altitude adapted ruminants amplifies parity-driven compositional shifts.

### 4.2. Covariance Structure of Milk Components: Correlation and Principal Component Feature Analysis

The present investigation employed multivariate statistical techniques to elucidate the covariance structure of fatty acid composition in yak milk. The results of the principal component analysis (PCA) indicated that the initial two principal components collectively accounted for 43.4% of the overall variance, effectively differentiating yak milk samples from various parities. PC1 was predominantly influenced by unsaturated fatty acids, including oleic acid and ARA, whereas PC2 was principally affected by saturated fatty acids, such as C15:0 and C21:0. This natural stratification, based on the degree of saturation of the fatty acids, reflects the existence of different metabolic pathways in the synthesis of yak milk fat and underscores parity’s systematic impact as a physiological determinant. These findings are in alignment with those of Wojciechowski and Barbano (2016), who identified the critical nature of fatty acid unsaturation for the differentiation of milk samples using mid-infrared spectroscopy [7]. This finding suggests that fatty acid saturation may have the capacity to serve as a universal biological marker that is capable of indicating the metabolic status of the mammary gland. However, this study uniquely employs this approach to yak milk from high-altitude plateaus, thereby addressing a significant research void. As reported by Currò et al. (2019) and Fan et al. (2023), research has been conducted on the impacts of the breed and lactation stage on goat milk fatty acid composition [13,36]. However, these studies did not thoroughly examine parity factors.

The biological mechanisms through which parity exerts its influence on the fatty acid composition are likely to be subject to multi-level regulation. The genetic correlations observed between medium-chain and saturated fatty acid groups, and milk fat percentage [39], are consistent with the results of our PCA loading distribution. Parity progression may involve altered mammary lipid synthesis enzyme expression patterns [40], with stable carbon isotope analysis supporting varying dietary fatty acid conversion efficiency [41]. It is evident that environmental and management factors also influence variability [42,43], but this must be understood about parity as a physiological condition. The present study provides a scientific foundation for enhancing yak milk quality whilst considering parity implications.

From a practical standpoint, the covariance architecture revealed by PCA offers an actionable framework for quality grading and product differentiation in the yak dairy sector. The clear separation of parity groups along PC1 (unsaturated fatty acid axis) and PC2 (saturated fatty acid axis) suggests that a simplified two-dimensional scoring system, derived from routinely measurable indicators, could be implemented at collection stations to classify incoming milk batches by their functional lipid profiles without recourse to full gas chromatographic analysis. Such classification would facilitate the allocation of milk with higher unsaturation indices toward premium functional dairy products (e.g., omega-3-enriched yoghurt or butter), while milk enriched in MCFAs could be directed toward energy-dense formulations. Additionally, the strong positive correlation between calcium and ARA identified in the correlation matrix (|r| > 0.67) has implications for nutritional labelling: calcium content, already a mandatory disclosure parameter on commercial labels, may serve as a proxy indicator for ARA status, thereby narrowing the information gap between the conventional and functional compositional data that are available to consumers.

### 4.3. Machine Learning-Driven Forecasting: A Cost-Effective and Innovative Technological Approach

The present investigation employed traditional milk components (fat, protein, VA, Na, and Ca) as independent variables to predict ARA, ALA, LA, and EPA levels using machine learning models, including XGBoost, GAM, SVM, and Random Forest (RF). All four models demonstrated noteworthy generalisation capabilities, aligning with prevailing dairy science trends that favour machine learning to enhance predictive accuracy. This methodology circumvents the limitations of feed-based fatty acid prediction models, such as those outlined by Daley et al. [9], which are constrained to controlled indoor feeding conditions.

Machine learning applications in the field of dairy nutrition have evolved from single-indicator forecasting to comprehensive intelligent decision-making. In the context of human donor milk, the integration of gradient boosting and elastic net methodologies has resulted in a substantial reduction in the mean absolute error (MAE) associated with predicting the protein and fat content [44]. Models synthesising routine milk composition with maternal and infant clinical data have achieved sensitivities and specificities over 85% [45]. Multimodal sensor data with time-series deep learning facilitates early detection of mastitis 48–72 h before clinical manifestation, achieving 92% prediction accuracy [46].

The accurate prediction of functional fatty acids necessitates the identification of intricate non-linear correlations. Mid-infrared spectroscopy (MIR) with machine learning offers high-throughput, cost-effective solutions for milk fatty acidomics. The MIR-RF and MIR-SVM methodologies have been shown to reduce analysis duration and material consumption by a factor of six while maintaining high levels of predictive precision [47,48]. Multi-model ensemble frameworks incorporating PLSR, elastic net, and convolutional neural networks have achieved concurrent prediction of milk protein subunits and processing attributes [48,49], thereby reinforcing the justification for model selection.

In practical terms, the machine learning models developed herein translate complex statistical outputs into operationally meaningful tools for the yak dairy industry. Specifically, the identification of calcium and protein as the two most influential predictors (importance scores of 0.45 and 0.27 in the ARA-XGBoost model, respectively) signifies that dairy processors and pastoral cooperatives only need to obtain two readily accessible and low-cost measurements—calcium via ICP-OES or ion-selective electrodes and protein via the Kjeldahl method or near-infrared spectroscopy—to generate reliable estimates of the ARA content in incoming milk. This eliminates the requirement for gas chromatographic fatty acid profiling, which demands expensive instrumentation, trained technicians, and extended analytical turnaround times that are impractical in the resource-constrained pastoral settings that are characteristic of the Qinghai-Tibetan Plateau. For smallholder yak herders, the deployment of these predictive models within a portable decision-support application could enable real-time milk quality assessment at the point of collection, facilitating differential pricing schemes that reward a higher functional fatty acid content and thereby incentivising herd management practices (e.g., preferential milking of third-parity animals) that maximise the PUFA yield.

Beyond individual farm-level applications, the scalability of these models holds significance for regional dairy industry governance. Regulatory authorities and dairy cooperatives could integrate the predictive algorithms into an existing milk quality monitoring infrastructure to establish a rapid screening system for functional fatty acid compliance. Such a system would support the development of origin-protected or quality-certified yak dairy product labels—analogous to the protected designation of origin (PDO) systems employed in European dairy sectors—that explicitly communicate the omega-3 and omega-6 PUFA advantages of high-altitude yak milk to consumers. Furthermore, in the context of breeding strategy formulation, the variable importance rankings generated by the machine learning models provide indirect guidance for trait prioritisation: the prominence of calcium as a predictor of ARA suggests that selection for enhanced mammary calcium transport capacity (e.g., through marker-assisted selection targeting calcium transport-related genes such as CaSR, whose regulatory role in mammary gland calcium secretion has been experimentally demonstrated) may simultaneously improve both the mineral and functional fatty acid profiles, thereby achieving pleiotropic breeding gains [50].

### 4.4. Molecular Docking: Elucidation of the Recognition Mechanisms of Bioactive Fatty Acids with Their Protein Targets

The present investigation employed AutoDock Vina for molecular docking analysis involving four bioactive fatty acids (LA, EPA, ALA, and ARA) with four lipid-associated protein targets (APOA1, APOA4, CD36, and FABP2). The integration of each ligand within the hydrophobic cavities was found to be stable, with the resultant binding energies ranging from −6.90 to −11.30 kcal mol^−1^. The docking configurations indicate that fatty acid carboxylate moieties are electrostatically attracted to positively charged residues at cavity entrances, followed by the establishment of hydrogen bonds with residues LEU-203, ALA-232, and LYS-206 in APOA1, or ARG-279, GLN-286, and GLY-280 in APOA4. The “carboxylate coordination coupled with hydrophobic-tail accommodation” model is consistent with crystallographic findings for the FABP family and AlphaFold structural predictions [51].

Proteins exhibit specific ligand affinities: CD36 has been observed to demonstrate a heightened response to long-chain polyunsaturated fatty acids, with EPA (−10.53 kcal mol^−1^) and ARA (−10.05 kcal mol^−1^) exhibiting stronger binding than LA or ALA, stabilised through hydrogen bonds with LYS-437. FABP2 demonstrates the highest EPA affinity of the studied molecules, with a binding constant of −11.30 kcal mol^−1^, anchored by TYR-15. The length of the fatty acid chains and the degree of unsaturation have been shown to collectively influence conformational flexibility. It has been demonstrated that increased double bonds facilitate optimal shape complementarity. This pattern provides a rationale for the observed prominence of EPA and ARA, which is consistent with epidemiological data associating omega-3 polyunsaturated fatty acids with cardiovascular protection [52,53]. These findings suggest potential therapeutic strategies that target lipid transport and metabolism [54].

The purpose of conducting these molecular docking simulations extends beyond theoretical mechanistic elucidation; the results bear direct relevance to both yak milk production optimisation and downstream technological applications. First, the differential binding affinities observed across the four protein targets provide a molecular-level rationale for understanding how the functional fatty acids that are enriched in third-parity yak milk are recognised and processed by key lipid metabolism proteins in the human body. The exceptionally strong EPA–FABP2 interaction (−11.30 kcal mol^−1^) suggests that the EPA present in yak milk is likely to be efficiently absorbed and transported in the intestinal epithelium, where FABP2 serves as the principal intracellular fatty acid carrier. This finding reinforces the nutritional superiority of EPA-enriched yak milk and provides a structural biological basis for marketing yak dairy products as functional foods with enhanced omega-3 bioavailability.

Second, the identification of specific binding residues (e.g., TYR-15 in FABP2, LYS-437 in CD36) opens avenues for targeted product development in the nutraceutical and pharmaceutical sectors. The knowledge of these key interaction sites enables the rational design of lipid-based delivery systems—such as nano-emulsions or liposomal formulations—that are optimised to preserve the structural integrity of EPA and ARA during dairy processing (pasteurisation, homogenisation, and spray-drying), thereby maximising their bioactivity upon consumption. For the yak milk industry specifically, these docking results suggest that processing protocols should minimise the thermal degradation of long-chain PUFAs, as the strong protein–ligand interactions demonstrated herein are contingent upon the preservation of native fatty acid conformations, particularly the cis-double bond geometry that governs shape complementarity within the hydrophobic binding pocket.

Third, from a comparative perspective, the binding energy hierarchy observed in the present study (EPA > ARA > LA > ALA for CD36 and FABP2) is consistent with the binding selectivity patterns of bovine beta-lactoglobulin for long-chain unsaturated fatty acids elucidated through flexible molecular docking [55], yet the absolute binding energies obtained herein are notably lower (i.e., stronger binding) than those reported for bovine milk protein–fatty acid complexes analysed by molecular dynamics and MMGBSA approaches [56]. This discrepancy may be attributable to the higher degree of unsaturation and the distinctive carbon chain length distribution that is characteristic of high-altitude yak milk fatty acids, which confer enhanced conformational flexibility and improved geometric complementarity within the binding cavities. Such comparative molecular evidence further substantiates the unique nutritional value proposition of yak milk as a high-altitude specialty dairy resource and supports the development of breed-specific and parity-specific quality standards that incorporate molecular interaction parameters as supplementary evaluation criteria.

## 5. Conclusions

This study integrates nutritional analysis, machine learning prediction, and molecular docking to explore lipid metabolism dynamics in yak milk across parities under plateau conditions. The results indicate that conventional milk composition indicators may potentially serve as candidate predictors for functional fatty acids, possibly offering a more accessible alternative to complex detection techniques. The preliminary identification of receptor binding sites may provide a structural reference for further investigation into milk-derived bioactive components. These findings may contribute to the nutritional evaluation of highland specialty dairy products and could inform future research directions, although independent model validation with larger and more diverse sample sets should be validated before broader application is considered.

## Figures and Tables

**Table 1:** F-test results of various indicators among different indicators (*p* < 0.05 only).

Abbreviation	Compounds	F	*p*	Ges
Ca	Calcium	5.369	0.012	0.318
FA1	C10:0 Capric acid	4.346	0.025	0.274
FA14	C20:4n6 Arachidonic acid	20.448	<0.001	0.64
FA15	C20:5n3 Eicosapentaenoic acid	9.878	<0.001	0.462
FA16	C21:0 Heneicosanoic acid	5.834	<0.001	0.337
FA2	C11:0 Undecanoic acid	4.288	0.026	0.272
FA25	C20:0 Arachidic acid	6.897	0.005	0.375
FA3	C12:0 Lauric acid	7.274	0.004	0.387
FA30	C17:0 Margaric acid	9.112	0.001	0.442
FA32	C18:3 Linolenic acid	9.472	<0.001	0.452
FA34	C18:0 Stearic acid	4.45	0.023	0.279
FA36	C16:0 Palmitic acid	5.324	0.013	0.316
FA5	C14:0 Myristic acid	16.953	<0.001	0.596
Na	Sodium	7.087	0.004	0.381
Pro	Protein	17.804	<0.001	0.608

**Table 2:** Top 20 significant correlations ranked by correlation coefficient.

Compound 1	Compound 2	Correlation
C15:0 Pentadecanoic acid	C22:0 Behenic acid	0.84
C21:0 Heneicosanoic acid	C15:0 Pentadecanoic acid	0.8
C22:0 Behenic acid	Vitamin A	0.76
C21:0 Heneicosanoic acid	C23:0 Tricosanoic acid	0.75
C21:0 Heneicosanoic acid	C20:0 Arachidic acid	0.75
C21:0 Heneicosanoic acid	C22:0 Behenic acid	0.74
C21:0 Heneicosanoic acid	C18:1 Oleic acid	−0.74
C20:0 Arachidic acid	C15:0 Pentadecanoic acid	0.73
C18:1 Oleic acid	C15:0 Pentadecanoic acid	−0.73
C15:0 Pentadecanoic acid	Vitamin A	0.73
Calcium	C20:4n6 Arachidonic acid	0.72
C21:0 Heneicosanoic acid	Vitamin A	0.71
C20:4n6 Arachidonic acid	C20:5n3 Eicosapentaenoic acid	0.71
C18:1 Oleic acid	Vitamin A	−0.69
C10:0 Capric acid	C12:0 Lauric acid	0.68
C12:0 Lauric acid	C17:0 Margaric acid	−0.68
C20:0 Arachidic acid	Vitamin A	0.67
C10:0 Capric acid	C6:0 Caproic acid	0.67
C18:2n6t Trans-linoleic acid	C14:0 Myristic acid	0.67
C20:0 Arachidic acid	C18:0 Stearic acid	0.67

**Table 3:** Summary table of optimal models.

Abbr1	Compounds	Model	RMSE	MAE	R_Squared	MAPE
FA14	ARA	XGBoost	0.022	0.019	0.769	14.446
FA15	EPA	Random Forest	0.011	0.008	0.478	11.865
FA32	ALA	GAM	0.097	0.081	0.76	5.076
FA33	LA	SVM	0.185	0.173	0.609	7.551

**Table 4:** Variable importance for the model.

	ARA~XGBoost	EPA~Random Forest	ALA~GAM	LA~SVM
Ca	0.449907	−0.000005	−0.001431	1.08211
FAT	0.013649	0.000011	0.013935	0.50441
Na	0.182968	0.00002	−0.006173	2.355952
Pro	0.27392	0.00001	−0.000411	0.458611
VA	0.079556	0.000014	−0.013497	2.294743

## Data Availability

The data presented in this study are available in this article and Supplementary Material.

## Supplementary Materials

The following supporting information can be downloaded at: https://www.mdpi.com/article/10.3390/ani16101477/s1, Table S1: Compounds and Their Abbreviations. Table S2: Names of All Tested Drugs. Table S3: F-test results of various indicators among different indicators.
